# Analysis of Plant Origin Antibiotics against Oral Bacterial Infections Using In Vitro and In Silico Techniques and Characterization of Active Constituents

**DOI:** 10.3390/antibiotics10121504

**Published:** 2021-12-08

**Authors:** Abdul Rafey, Adnan Amin, Muhammad Kamran, Uzma Haroon, Kainat Farooq, Kenn Foubert, Luc Pieters

**Affiliations:** 1 NPRL, Gomal Centre of Pharmaceutical Sciences, Faculty of Pharmacy, Gomal University, Dera Ismail Khan 29050, Pakistan; abdulrafe4667@gmail.com (A.R.); adnan.amin@gu.edu.pk (A.A.); m.kamran.gu@gmail.com (M.K.); 2 Department of Dentistry, D.H.Q Teaching Hospital, Dera Ismail Khan 29050, Pakistan; uzma.haroonDHQ@gmail.com; 3 Sardar Begum Dental College, Ghandhara University, Peshawar 25000, Pakistan; kainat.farooq97@gmail.com; 4 Natural Products & Food Research and Analysis (NatuRA), Department of Pharmaceutical Sciences, University of Antwerp, Universiteitsplein 1, 2610 Antwerp, Belgium; kenn.foubert@uantwerpen.be

**Keywords:** Ayurveda, medicinal plants, biofilm, oral cavity, diabetes mellitus, quorum-sensing, dental plaque

## Abstract

The pervasiveness of oral bacterial infections in diabetic patients is a serious health concern that may produce severe complications. We investigated 26 Ayurvedic medicinal plants traditionally used for treatment of the oral bacterial infections with the aim to look for new promising drug leads that can be further employed for herbal formulation design. The plants were grouped into three categories based on traditional usage. All plant extracts were examined for antibacterial, antibiofilm and antiquorum-sensing properties. The plants with significant activities including *Juglans regia*, *Syzygium aromaticum*, *Eruca sativa*, *Myristica fragrans*, *Punica granatum* and *Azadirachta indica* were further analyzed using HPLC-DAD-QToF and GC-MS. In silico and in vitro activity was evaluated for selected constituents. Finally, it could be concluded that eugenol and 2-phenylethylisothiocyanate are major contributors towards inhibition of bacterial biofilms and quorum sensing.

## 1. Introduction

The oral bacterial infections include a wide array of inflammatory conditions that upset various auxiliary structures of the teeth including the periodontal ligament, gingiva and bone. This condition finally leads to loss of teeth with systemic inflammation as seen in periodontitis [1]. Most of isolated bacteria isolated from oral cavity belong to the genera *Prophyromonas*, *Enterococcus*, *Campylobacter*, *Bacteroides*, *Streptococcus*, *Actinomyces*, *Staphylococcus*, *Eubacterium*, *Lactobacillus*, *Leptotrichia*, *Treponema* and *Fusobacterium* [2,3] Some common bacteria isolated from oral cavity include *Staphylococcus epidermidis*, *Staphylococcus aureus* (dental plaque) [4,5] *Fusobacterium nucleatum*, *Streptococcus mutanTannerella forsythia*, *Fusobacterium* spp., *Prevotella nigrescens*, *Prevotella intermedia*, and *Porphyromonas gingivalis* [6,7] More importantly, significant changes in the oral environment like orthodontic appliances, pH changes, antibiotics usage, diabetes etc. upset the microbial homeostasis that may lead to periodontitis and dental caries [8].

Diabetes mellitus is a predominant systemic disease that influences periodontitis to a great extent [9]. Existing statistics provide a strong indication towards interactions of diabetes, gingivitis and periodontitis. The oral cavity possesses an exclusive environment which generally facilitates the growth of an array of microorganisms by providing a diverse supply of nutrients, oxygen and humidity [10]. The presence of soft (gingiva) and non-shedding hard (teeth) tissues offers a prospective site for adherence and consequent contact of microorganisms with various host cells [11]. Just after cleaning the teeth, the natural fluid present in the oral cavity causes adsorption of a thin acquired pellicle (composed of saliva glycoproteins including statherin, α-amylase, proline-rich proteins, agglutinin and mucins) [12], which induces a change in free energy and surface charge that facilitates bacterial adherence to produce biofilms [13]. The biofilm mass increases by a continuous growth and consequent adsorption of other bacterial species through co-aggregation. It is well documented that pathogens possess a quorum-sensing (QS) controlled ability to form biofilms, which leads to severe infections [14]. QS is termed as the ability of bacteria to perceive and respond rapidly to changes in cell density [15], with the help of small secreted signaling molecules known as auto-inducers. Several QS-based factors, such as bio-surfactant production, exopolysaccharide (EPS) production, swimming and swarming motility have been associated with biofilm formation.

Plant-derived compounds have a long history of treating microbial infections [16] and have received ample attention as a source of anti-QS compounds for the inhibition of biofilms [17]. In traditional medicinal systems including Ayurveda, medicinal plants like *Azadirachta indica*, *Juglans regia*, *Salvadora persica*, *Syzygium aromaticum Myristica fragrans* and *Punica granatum* (Table 1) are used that have a long history of medical use in humans. Such medicinal plants or their formulations are being also explored for the purpose of controlling biofilms, due to their non-toxic nature [18].

In current times, in silico tools are commonly used for prediction of drug binding to target site. Generally, drug leads are found using a docking algorithm that helps to classify the optimal binding mode of a drug at the active site of a target molecule. The transcriptional regulators 2Q0J and 3QP1 are used for bacterial biofilm and Quorum sensing in molecular docking analysis to predict possible binding affinities of constituents. This project mainly focuses on the assessment of Ayurvedic medicinal plants for antiquorum-sensing and biofilm-inhibiting properties towards clinical strains isolated from diabetic patients with periodontal disease, and characterization of their active constituents. We intended to use the findings of this project for the development of herbal formulation.

## 2. Materials and Methods

### 2.1. Bacterial Strains, Growth Media and Chemicals

The bacteria isolated from dental plaques were identified as *Staphylococcus epidermidis* and *Staphylococcus aureus* (Specimen deposited in Pakistan culture bank), whereas the commercial strains used during investigation included *Staphylococcus aureus* (ATCC 33862), *Chromobacterium violaceum* (DSM 30191) and *Pseudomonas aeruginosa* (ATCC 15442). The bacterial growth media used included, Tryptic Soya Broth (TSB), nutrient agar (Hi Media, India) Luria-Bertani Broth (LB) (Oxoid). The standard compounds were purchased commercially, including juglone (Santa Cruz Biotechnology, Santa Cruz, CA, USA), eugenol (Fluka Honeywell, Seelze, Germany), quercetin (Sigma Aldrich, St. Louis, MO, USA), *trans*-caryophyllene (Fluka Honeywell, Seelze, Germany), ciprofloxacin (Sigma Aldrich, St. Louis, MO, USA) and azithromycin (Sigma Aldrich, St. Louis, MO, USA), quercitrin (Sigma Aldrich, Steinheim, Germany), 2-phenylisothiocyanate (Sigma Aldrich, Steinheim, Germany), α-humulene (Extrasynthese, Genay, France), caryophyllene-oxide (Fluka Honeywell, Seelze, Germany) and apigenin (Sigma Aldrich, Steinheim, Germany).

### 2.2. Isolation and Sequencing of Bacteria

The approval from Ethical review board, Gomal University, D.I. Khan was obtained (2019). Ten patients with periodontitis were enrolled in the investigation and dentist obtained dental plaques (with informed consent) using sterilized techniques. Bacteria were isolated and purified using standard microbiological techniques and biofilm production was initially checked using Congo red agar method [45]. The PCR-based identification was performed in National Culture Collection of Pakistan (NCCP) using 16S rRNA gene sequencing [46]. A modified method [47] (Supplementary Materials) was used for genomic DNA extraction from bacteria, followed by agarose gel electrophoresis and PCR (polymerase chain reaction) [48] (Supplementary Materials) was used. The genes alignment was performed for exact match with NCBI nucleotide database by using nBLAST (Supplementary Materials) and strains were identified as *Staphylococcus aureus* and *Staphylococcus epidermidis*. The antibacterial activity of all selected plants was checked against both *Staphylococos epidermidis* and *Staphylococcus aureus* (clinical isolates) and *Pseudomonas aureginosa* and *Staphylococos aureus* (ATCC strains).

### 2.3. Plant Material

Plant species investigated here are listed in Table 1. Plant material was obtained from authorized herbal medicine stores. The extracts were prepared by cold maceration (90% methanol) for 10 days (repeated three times) and further liquid—liquid fractionation was accomplished using a rotary evaporator (40 °C). The dried plant material was stored at −4 °C until further usage. Contrarily, the essential oils were obtained using a Clevenger type apparatus (hydro-distillation) and fixed oils were obtained using the cold press method.

### 2.4. Chromatographic Analysis

#### 2.4.1. HPLC-DAD Analysis

A detailed HPLC analysis of extracts was performed using Agilent^®^ 1200 series system (HPLC-DAD, Agilent Technologies, Santa Clara, CA, USA). The samples (10 μL) were injected at a flow rate of 1 mL/min with acetonitrile: water (0.1% formic acid) gradient (5% Acetonitrile to 100% in 40 min). Samples were prepared in a concentration range from 1 to 10 mg/mL in methanol. A Phenomenex Luna C18 column (silica-based, 250 × 4.6 mm, 5 μm) (Phenomenex, Torrence, CA, USA) was used.

#### 2.4.2. HPLC-DAD-QToF Analysis

The MS^2^ analysis of plant extracts was performed by using QToF spectrometer (Xevo G2-XS QToF spectrometer, Waters, Milford, MA, USA), coupled with a LC system (Acquity). The samples were injected (5 μL) with a flow rate of 0.6 mL/min using a reverse phase column (BEH Shield, 100 mm × 2.10 mm, 1.7 μm, Waters, Milford, MA, USA). A gradient (40 min) mobile phase (H_2_O + 0.1% formic acid and Acetonitrile + 0.1% FA) was used. The DAD spectra were recorded between 190 and 500 nm. A full scan data (ESI [−] and ESI [+], *m/z* 50 to 1500) was recorded using sensitivity mode (approximate resolution: 22,000 FWHM) during first scan. Other parameters were source, desolvation temperature at 120 °C and 550 °C, spray voltage was +1.0 kV and −0.8 kV; cone gas flow 50.0 L/h, and desolvation gas flow 1000.0 L/h, respectively.

#### 2.4.3. GC-MS Analysis

The essential oil analysis was accomplished by using GC-MS (Thermo Scientific, WM, USA) with an (AOC-20i) and FID detector (flame ionization detector). The sample was injected (0.5 μL) and Helium (1 mL/min) was used as carrier gas. The DB-5 MS capillary column with (30 m × 0.25 mm) with 0.25 μm film thickness was used. The column oven temperature was initially set at 40–90 °C (2 °C/min) that was upraised to 90–240 °C (3 °C/min). The temperature of injector and detector was kept constant at 240 °C and 280 °C, correspondingly. The Trace GC (Ultra DSQ) was used for identification in electron ionization mode operating at 70 eV. The mass units were monitored from 35 to 500 amu. A capillary column (AT-5MS, Grace) dimensions 30 m × 0.25 mm with 0.25 μm film thickness was used using same temperature conditions as GC analysis. The components identification was performed using comparison with NIST library.

### 2.5. Molecular Docking

The molecular docking studies were performed using AutoDock vina v 4.2. In the first part, structures (X-ray crystallographic) of the transcriptional regulators 2Q0J [49] and 3QP1 [50] were downloaded from the Protein Data Bank (PDB). In subsequent part, the 3D structures of test samples (SDF format) were obtained from Pubchem database. Then the PDB format files were created by using Accelrys DS Visualizer 2.0 [51]. The active pocket dimensions for targets were recorded by using CASTp 3.0, followed by compulsory processing for removal of water molecules and H atom, charges addition. Finally, molecular docking was performed using Lamarckian Genetic Algorithm embedded in AutoDock v 4.2. [52]. A total number of 45 poses were generated and grouped according to their RMSD and best docked molecules [ΔG] were analyzed using Ligplot^+^ Accelrys DS Visualizer 2.0 and PYMOL.

### 2.6. Biological Activities

#### 2.6.1. Determination of MIC and MBC (Minimum Inhibitory and Bactericidal Concentrations)

The antimicrobial assays were performed with the help of a modified protocol [53]. To the 96 microwell plates (NEST, China), 24 h old bacterial cultures (50 µL, 1.5 × 10^7^ CFU/mL) and test sample (50 µL, various dilutions) were added. The plates were then incubated at 37 °C for 24 h and afterwards resazurin (40 µL, 0.015%) solution was added and incubated further for 60 min (37 °C). A 96-microplate reader (Hippo MPP-96, Biosan) was used for recording results. For MBC determination, 10 μL of bacterial suspensions was collected from MIC microwells and streaked on Muller Hinton agar plates and incubated for 24 h at 37 °C. The growth on Muller Hinton agar plates was monitored by counting colonies. Ciprofloxacin was used as reference standard.

#### 2.6.2. Antibiofilm Activity

A modified method for inhibition of biofilm formation was used during this study [54]. Briefly, 24 hrs old bacterial cultures in TSB media (adjusted with 0.5 McFarland) and 100 μL of test sample (0.01–3 mg/mL) were added to 12-well polystyrene plates and incubated for 24 h at 37 °C. Afterwards, the cell growth in the plates was monitored at 592 nm. For quantification, the crystal violet staining of biofilms (in 12-well plates) was performed, followed by addition of 95% ethanol to stained cells. Finally, the absorbance (Abs) was recorded at 592 nm for quantification of biofilm formation. The % inhibition was calculated using the following formula:

% Inhibition = [1 − (OD of test sample/OD of control)] × 100

#### 2.6.3. Antiquorum-Sensing

The anti QS potential of compounds was determined by using the previously described procedure [55]. The BHIA (Brain Heart Infusion Agar) was inoculated with an overnight bacterial culture (*C. violaceum*, 1/100 ratio) followed by placing sterilized paper discs (6 mm). The test sample (12 µL, 0.01–3 mg/mL) was added on each disc and kept to dry for 40 min. Later, the seeded plates were incubated at 30 °C for 3 days. The results were determined by recording the zone of inhibition around discs. Ciprofloxacin was used as reference drug.

#### 2.6.4. Violacein Inhibition Assay

For quantification of violacein produced by *C. violaceum*, an improved procedure was used [56]. Briefly, an overnight grown culture (200 μL) of *C. violaceum* (OD = 0.4 at 600 nm) was added to sterilized microtiter plates containing various concentrations of test samples (1–4 mg/mL). The plates were kept at 30 °C for 24 h and monitored for inhibition of violacein production by observing the absorbance at 585 nm. The % of inhibition was calculated by using the following formula:

% Violacein inhibition = [1 − (OD test sample/OD control)] × 100

## 3. Results and Discussion

### 3.1. Screening of Plant Extracts

Initially a detailed survey of Ayurvedic medicinal plants reported to possess either antibacterial or antibiofilm activity, or used for oral cavity problems, was performed. A diverse collection of traditional medicinal plants was employed. The plants were categorized as category A (miswak or chewing sticks, commonly employed for oral hygiene), category B (oils, that have been used for oil pulling in traditional Ayurveda) and category C (traditional antimicrobial plants that have Ayurvedic claims for rinsing of the oral cavity) (Table 1). Initially the biofilm producing strains were tested against commonly prescribed antibiotics and antibiograms were recorded (Table 2). It was clear from these results that most of the tested antibiotics were resistant to clinical strains.

In plant species from category A, in general the better inhibition against *S. aureus* (ATCC) with significant MIC values (ranging from 0.012 to 0.097 mg/mL) (Table 3) was observed. Overall, the *Juglans regia* extracts (root peel and stem peel) were able to produce significant inhibition (0.048–0.78 mg/mL) against the clinical isolate of *S. aureus*. Likewise, the category A plants also showed better inhibition against *Pseudomonas aureginosa* (ATCC) at diverse MIC values (ranging from 0.024–1.2 mg/mL). In the case of *S. epidermidis* (clinical isolate), most plant extracts showed no inhibition; however, the *Juglans regia* extract presented significant inhibition (0.048–0.78 mg/mL). During initial anti-biofilm analysis, *Juglans regia* extracts showed a moderate inhibition (52% at 200 µg/mL) (Table 4). All other plant species from category A either showed no inhibition or presented low levels of inhibition (<50%). Based on the antibacterial properties, the plant species with significant activity were further processed in anti-biofilm and anti-QS assays. A moderate anti-QS activity (5–7 mm zone of inhibition and 51% inhibition of violacin) was noticed for *Juglans regia* extracts. These results are in compliance with the obtained MICs. Literature has suggested that active ingredients present in *Juglans regia* may include juglone, regiolone, glansreginin A, glansreginin B and proceroside [57,58,59] (Table 5), that may be contributing to inhibition.

In plant species from category B (fixed and essential oils), only *Syzygium aromaticum* (0.024–0.097 mg/mL) showed promising inhibition against both clinical and reference strains (ATCC) (Table 2). The antibiofilm results reveal the highest inhibition by *Syzygium aromaticum* (72% at 0.19%) (Table 4). The *Syzygium aromaticum* oil showed an excellent inhibition (16 mm) of *C. violaceum* with a high (61%) violacein inhibition at the tested concentration (Table 4). A detailed literature review has suggested that *Syzygium aromaticum* oil mainly contains eugenol, caryophyllene, caryophyllene-oxide, eugenyl acetate and α-humulene [11,69]. Thus, the strong inhibition could possibly be due to a single ingredient or due to an additive or synergistic effect. Eugenol and α-humulene have been reported to show strong antimicrobial properties [69]. The *Eruca sativa* oil (0.024–0.097 mg/mL) also showed promising inhibition against both clinical and reference strains (ATCC) (Table 3). Additionally, moderate antibiofilm activities (58%) and anti-QS (14 mm zone of inhibition, 52% inhibition of violacein) were recorded in the case of *Eruca sativa* oil (Table 3). The main components of *Eruca sativa* oil are bis-[4-isothiocyanatobutyl) disulphide, sulforaphane, 2-phenylethyl isothiocyanate, 3-butenyl isothiocyanate and erucic acid [14,61] (Table 5), that may be contributing towards strong inhibition.

In plant species from category C (traditional plants with antimicrobial claims), the majority of the tested extracts were not able to show any reportable activity (maximum tested concentration 1.2 mg/mL); however the *Myristica fragrans* extracts (both seeds and mace) showed remarkable activity (MIC ranging from 0.024 to 0.097 mg/mL) (Table 3). With regard to antibiofilm activity, a mild activity was noticed (35–39%). Additionally, similar trends were seen in anti-QS activity (2 mm zone of inhibition; 32–35% inhibition of violacein). Researchers have reported that *Myristica fragrans* is a rich source of sabinene, elemicin, caryophyllene, caryophyllene-oxide, α-phellandrene, α-pinene and β-pinene [66,67] (Table 3). Sabinene and elemicin have strong antibacterial properties [68] that can contribute to the observed activity. Likewise, *Illicium verum* extracts also showed significant activity (0.048–1.2 mg/mL). However, a mild level of antibiofilm and anti QS activity was recorded (Table 3). The essential oil *Illicium verum* mainly contains *trans*-anethol, feniculin [62], estragole, limonene and 4-allylanisole [64] (Table 4). Based on relative abundance, it was concluded that *trans*-anethol may contribute to the antimicrobial activity [64].

The peel extracts of *Punica granatum* also showed strong inhibition against all tested strains (MIC ranging from 0.19–0.78 mg/mL) (Table 3). During antibiofilm activity, a moderate inhibition was noticed (55%). Similar trends were seen in anti-QS activity (5 mm zone of inhibition; 52% inhibition of violacein). The peel is commonly known for strong antioxidant properties and is a rich source of punicalagin, punicalin, apigenin, quercetin, kaempferol, gallic acid and ellagic acid [59,70] (Table 5). The presence of phenolics and flavonoids may contribute to strong antibacterial properties.

Lastly, *Azadirachta indica* seed oil also showed significant antimicrobial (0.09–0.78%) antibiofilm (54%) and antiquorum-sensing activities (6 mm). The *A. indica* seed oil has been reported to contain nimbidine, azadirachtin (Azadirachtin A), salannol, salannin [60], which possess antimicrobial properties [71]. In our case, the major components were not tested due to the unavailability of compounds.

### 3.2. GC-MS and HPLC-DAD-MS-QToF Analysis

Based on preliminary analysis, the plant samples with significant antibacterial activity were analyzed using HPLC-DAD. Afterwards GC-MS (oils) and HPLC-DAD-MS-QToF analysis (crude extracts) was performed covering all active plants. Chromatographic profiles are available as Supplementary Materials (Figures S1–S13). The results were compared with literature (Table 5), and pure available compounds were screened further to predict their role in the observed activities of plant extracts or oils.

### 3.3. Molecular Docking of Compounds

**Figure 1:**
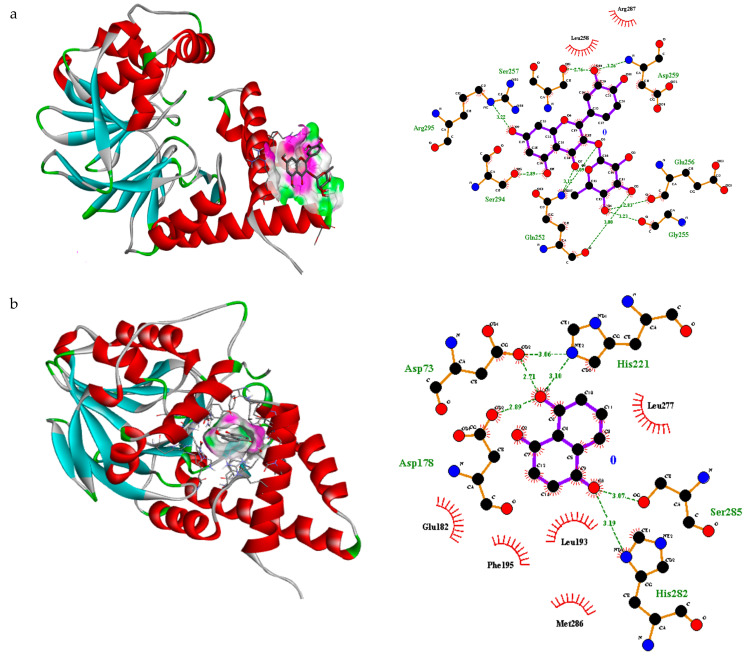

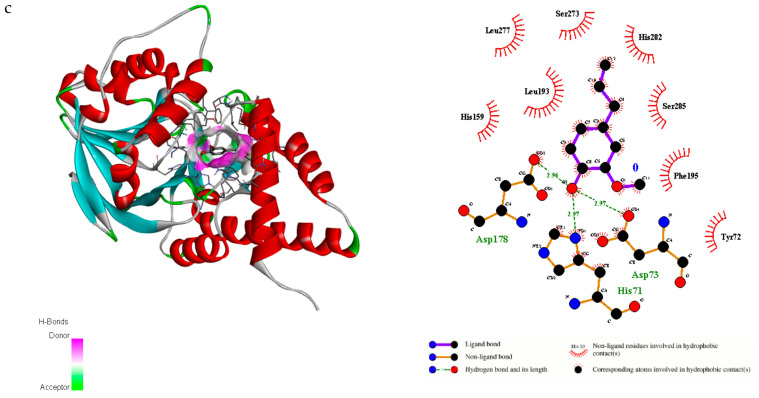
3D interaction and H, non-H bonding interactions of (**a**) querccitrin (pose 1) (**b**) juglone (pose 1) and (**c**) eugenol (pose 2) inside binding sites of transcriptional regulator 2Q0J.

In the transcriptional regulator 2Q0J, quercitrin showed a nice fit in the binding pocket with pose 9 and free binding energy −6.6 ΔG (kJ mol^−1^) (Table S10). Quercitrin showed strong H-bonding interaction with seven amino acid residues including Asp259, Glu256, Gly255, Gln252, Ser294, Arg295, Ser257 (Figure 1). Likewise, juglone presented a good fitting in the binding pocket with pose 1 and free binding energy −8.5 ΔG (kJ mol^−1^) (Table S10). Juglone displayed strong H-bonding interaction with five amino acid residues including Asp73, Asp 178, His221, His 282, Ser 285 (Figure 1). Likewise, eugenol displayed a good placement in the binding pocket with pose 2 with free binding energy −6.2 ΔG (kJ mol^−1^) (Table S10). A strong H-bonding interaction with three amino acid residues including Asp73, His71, Asp178 was observed (Figure 1). Interestingly, *trans*-caryophyllene and α-humulene did not present any H-bonding interactions within the prescribed pocket (Table S10). It was therefore concluded that quercitrin, juglone and eugenol may be contributing towards biological activity.

**Figure 2:**
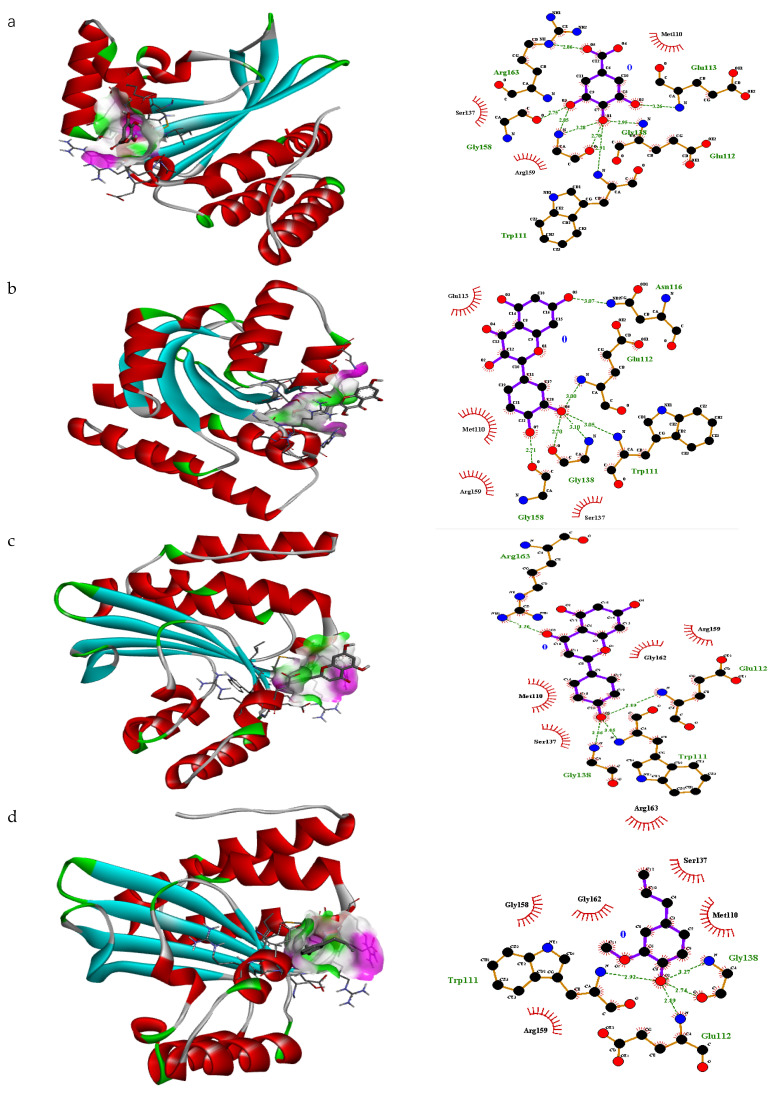
3D interaction and H, non-H bonding interactions of (**a**) gallic acid (pose 1), (**b**) quercitrin (pose 4), (**c**) apigenin (pose 3) and (**d**) eugenol (pose 6) inside binding sites of transcriptional regulator 3QP1 with pose rank 6.

In the transcriptional regulator 3QP1, gallic acid showed the best fitting (free binding energy −5.1 ΔG kJ mol^−1^) in the binding pocket of this protein with pose rank 1 (Figure 2). The H-bonding interacting residues included Glu113, Glu112, Gly138, Trp111, Gly158 and Arg163 Similarly, quercitrin showed a nice fit in the binding pocket with pose 4 with free binding energy −7.2 ΔG (kJ mol^−1^) (Table S10). Quercitrin showed strong H-bonding interaction with six amino acid residues including Arg59, Gly136, Ser137, Pro52, Glu160 and Arg163 (Figure 2). Likewise, apigenin showed an acceptable arrangement in the binding pocket with pose 3 with free binding energy −6.4 ΔG (kJ mol^−1^) (Table S10). In this case, strong H-bonding interactions were noticed with four amino acid residues including Glu112, Trp111, Gly128, Arg163 (Figure 2). In the case of eugenol pose 6 (free binding energy −5.0 ΔG (kJ mol^−1^) (Table S10) showed a nice fit in the binding pocket of transcriptional regulator 3QP1. A strong H-bonding interaction with three amino acid residues including Trp111, Gly128 and Glu112 was recorded (Figure 2). *Trans*-caryophyllene and α-humulin were unable to present any interaction within the prescribed pocket (Table S10). Thus, it was hypothesized that gallic acid, quercitrin, apigenin and eugenol may contribute towards significant inhibition. Based on docking results of these compounds against all targets it was concluded that the compounds with more H-bonding interactions may contribute towards significant biological activities. Therefore, based on docking results, in vitro analysis of these compounds was performed.

### 3.4. Biological Activities of Pure Compounds

The major components of *Syzygium aromaticum* oil include eugenol, caryophyllene, caryophyllene-oxide and α-humulene (Table 5). During antibacterial analysis, a reportable inhibition was recorded against all strains (MIC ranging from 0.0003 to 0.0.39 mg/mL) (Table 6). A significant inhibition of bacterial biofilms (52–55%) and quorum-sensing (15 mm zone of inhibition and 57.2% violacein inhibition) was observed (Table 7). Similarly, caryophyllene-oxide showed antibacterial activity (MIC range 0.05–0.75 mg/mL) against all tested bacterial strains (Table 6). During antibiofilm assays, poor inhibition (17%) was recorded against *S. epidermidis* (clinical strain), whereas a moderate inhibition (51%) was observed against *S. aureus* (clinical strain). However, a moderate inhibition of quorum-sensing (4 mm zone of inhibition; 54% violacein inhibition) was recorded (Table 7). In case of α-humulene a moderate antibacterial activity was seen (0.03–0.18 mg/mL) against all tested strains (Table 6). Similarly, a moderate (56–58%) antibiofilm activity was recorded against tested strains and only a slight anti-QS activity (1 mm zone of inhibition; 40% violacein inhibition) was noticed (Table 7).

The major constituents of *Juglans regia* bark include quercitrin, quercetin and juglone (Table 5). The juglone showed significant inhibition (MIC range 0.005–0.075 mg/mL) (Table 6); however, during antibiofilm and anti-QS sensing activities, no inhibition was recorded at the tested concentrations (Table 7). Quercetin showed a moderate inhibition of tested strains (MIC range 0.375–1.25 mg/mL), and did not show any inhibition of the clinical strain *S. aureus*. Finally, although quercitrin showed very good docking results (Table S1), due to unavailability of this component, we were unable to test it. It was therefore concluded that components of *Juglans regia* bark may act synergistically towards the inhibition shown by the *J. regia* extract.

With regard to *Eruca sativa* oil, 2-phenylethylisothiocyanate was evaluated for antibacterial activity and inhibition was recorded (MIC range 0.005–0.18 mg/mL) (Table 6). Interestingly, it showed a significant inhibition of biofilm formation (57%) and anti-QS activities (9 mm zone of inhibition; 56% violacein inhibition) (Table 7).

The *Punica granatum* peel is a rich source of diverse polyphenols (Table 5), and strong antioxidant and antimicrobial properties have been reported. Additionally, during our preliminary investigations, peel extracts showed moderate antimicrobial activities (Table 3). The polyphenols including gallic acid, ellagic acid, quercetin and apigenin were tested for antimicrobial, antibiofilm and anti-QS properties. All tested compounds showed a moderate inhibition (MIC range 0.125–0.625 mg/mL). Only apigenin inhibited all tested strains (Table 6). Contrarily, none of the compounds showed inhibition of quorum-sensing or antibiofilm properties at the tested concentrations (Table 7).

Likewise in *Myristica fragrans*, tested compounds included caryophyllene, caryophyllene-oxide and α-humulene, which were discussed above. Lastly, *Azadirachta indica* seed oil also showed significant antimicrobial (0.09–0.78%), antibiofilm (58%) and anti-QS-sensing activities (14 mm zone of inhibition; 52% violacein inhibition) as discussed earlier. However, the major components were not tested due to unavailability of these compounds.

## 4. Conclusions

It was concluded that *Syzygium aromaticum*, *Juglans regia*, *Eruca sativa*, *Punica granatum* and *Azadirachta indica* possessed antibacterial, antibiofilm and antiquorum-sensing properties. We therefore propose the use of these plant extracts or oils in formulations for the maintenance of oral hygiene as mentioned in ancient Ayurvedic literature.

## Figures and Tables

**Table 1:** Traditional uses of selected Ayurvedic medicinal plants.

Plant Species	Ayurvedic/Traditional Usage	References
Category A (Chewing sticks)		
*Azadirachta indica* A. Juss	Antibacterial, antibiofilm	[19]
*Olea europaea* L.	Antibacterial, antibiofilm	[20]
*Citrullus colocynthis* (L.) Schrad.	Antibacterial	[21]
*Echinops echinatus* Roxb.	Antibacterial,	[22]
*Juglans regia* L. ^1^	Antibacterial	[23,24]
*Juglans regia* L. ^2^	Antibacterial, Stem and bark used for teeth cleaning as chewing sticks in Pakistan and India	[23,24]
*Salvadora persica* L. ^3^	Antibacterial, antidiabetic	[19,25]
*Salvadora persica* L. ^4^	Antibacterial, antidiabetic	[19,25]
Category B (Oils)		
*Azadirachta indica* A. Juss	Antibacterial, antibiofilm	[26]
*Brassica nigra* (L.) K. Koch	For oil pulling	[27]
*Eruca sativa* Mill.	For oil pulling	[28]
*Lagenaria siceraria* (Molina) Standl.	For oil pulling	Local trad. use
*Phyllanthus emblica* L.	For oil pulling	[29]
*Prunus dulcis* Mill. ex Rchb.	For oil pulling	[30,31]
*Psoralea corylifolia* L.	For oil pulling	[32]
*Syzygium aromaticum* (L.) Merr. & L.M. Perry	Antibacterial	[33]
Category C (extracts)		
*Allium sativum* L. ^5^	Antibacterial, antibiofilm	[34]
*Allium sativum* L. ^6^	Antibacterial, antibiofilm	[35]
*Anacyclus pyrethrum* (L.) Lag	Antibacterial, antibiofilm	[36]
*Calotropis procera* (Aiton) Dryand	Antibacterial, antibiofilm	[37]
*Centella asiatica* (L.) Urb	Antibacterial, antibiofilm	[38]
*Illicium verum* Hook.f.	Antibacterial, antibiofilm	[39]
*Myristica fragrans* Houtt. ^7^	Antibacterial, antibiofilm	[40]
*Myristica fragrans* Houtt. ^8^	Antibacterial, antibiofilm	[40]
*Punica granatum* L.	Antibacterial, antibiofilm	[41]
*Terminalia arjuna* (Roxb. ex DC) Wight & Arn.	Antibacterial, antibiofilm	[42]
*Urtica dioica* L.	Antibacterial, antibiofilm	[43]
*Wrightia tinctoria* R.Br.	Antibacterial, antibiofilm	[44]

^1^ Stem peel; ^2^ root peel; ^3^ origin Saudi Arabia; ^4^ origin Pakistan (different origins were screened because of local believes); ^5^ black garlic; ^6^ garlic; ^7^ seed; ^8^ mace.

**Table 2:** Antibiogram profiles of tested strains.

Bacterial Strain	Ciprofloxacin	Moxifloxacin	Gentamicin	Ceftriaxone	Ceftazidime	Cefipime	Amoxicillin	Coamoxiclavev	Imipenem	Meropenem	Azteronam	Metronidazole	Azithromycin	Ofloxacin	Tetracycline
*Staphylococcus aureus ^1^*	20	80	100	100	100	100	90	80	60	60	100	100	100	90	90
*Staphylococcus epidermidis ^1^*	20	90	100	100	100	100	100	80	60	60	100	100	100	100	100
*Staphylococcus aureus ^2^*	10	0	60	40	40	20	30	10	0	0	100	100	0	40	40
*Pseudomonas aureginosa ^2^*	10	0	100	40	40	20	40	10	0	0	100	100	0	50	30

^1^ = clinical strain, ^2^ = standard strain.

**Table 3:** Determination of MIC values (mg/mL) of tested plant species.

Plant Species	*S. aureus* ^a^	*S. epidermidis* ^b^	*S. aureus* ^c^	*P. aerugionasa* ^d^
*Allium sativum* ^1^	>100	>100	>100	1.2
*Allium sativum* ^2^	>100	>100	0.6	0.6
*Anacyclus pyrethrum*	>100	1.2	>100	1.2
*Azadirachta indica*	>100	0.78	0.024	0.024
*Azadirachta indica* oil	0.39	>100	0.09	0.78
*Brassica nigra* oil	>100	>100	>100	>100
*Calotropis procera*	>100	>100	1.2	1.2
*Centella asiatica*	>100	>100	1.2	0.048
*Citrullus colocynthis*	>100	>100	0.012	>100
*Echinops echinatu*	>100	>100	0.024	>100
*Eruca sativa* oil	0.012	0.097	0.024	0.012
*Illicium verum*	1.2	0.6	0.048	0.097
*Juglans regia* ^3^	0.78	0.048	0.097	0.048
*Juglans regia* ^4^	0.048	0.78	0.097	0.097
*Lagenaria siceraria* oil	>100	>100	>100	>100
*Myristica fragrans* ^5^	0.097	0.024	0.097	0.024
*Myristica fragrans* ^6^	0.097	0.048	0.78	0.048
*Olea europaea*	>100	1.2	0.048	0.048
*Phyllanthus emblica* oil	>100	>100	>100	>100
*Prunus dulcis* oil	>100	>100	>100	>100
*Psoralea corylifolia* oil	>100	>100	>100	>100
*Punica granatum*	0.78	0.39	0.19	0.39
*Salvadora persica* ^7^	>100	>100	0.024	1.2
*Salvadora persica* ^8^	>100	1.2	0.012	1.2
*Sesamum radiatum*	>100	>100	>100	>100
*Syzygium aromaticum* oil	0.024	0.097	0.024	0.024
*Terminalia arjuna*	>100	>100	0.78	0.78
*Urtica dioica*	>100	>100	>100	1.2
*Wrightia tinctoria*	>100	>100	0.78	1.56
Ciprofloxacin	< 0.004	< 0.004	0.009	0.009
Azithromycin	>100	>100	0.008	0.002

^1^ Black garlic; ^2^ garlic; ^3^ stem peel; ^4^ root peel; ^5^ seed; ^6^ mace; ^7^ origin Saudi Arabia; ^8^ Origin Pakistan; ^a^ isolated strain; ^b^ isolated strain; ^c^ ATCC 33862; ^d^ ATCC 15442;

**Table 4:** Antibiofilm and antiquorum-sensing activity of plant extracts and essential/fixed oils.

Plant Species	Inhibition of Bacterial Biofilm *	Zone of Inhibition **	Violacein Inhibition
*Allium sativum* ^1^	Nil ^a^	0 ^a^	Nil ^a^
*Allium sativum* ^2^	Nil ^a^	0 ^a^	Nil ^a^
*Anacyclus pyrethrum*	Nil ^a^	0 ^a^	Nil ^a^
*Azadirachta indica*	Nil ^a^	0 ^a^	Nil ^a^
*Azadirachta indica* oil	54% ^a^	6 ^a^	54% ^a^
*Brassica nigra* oil	Nil ^a^	0 ^a^	Nil ^a^
*Calotropis procera*	25% ^a^	0 ^a^	Nil ^a^
*Centella asiatica*	28% ^a^	2 ^a^	26% ^a^
*Citrullus colocynthis*	Nil ^a^	0 ^a^	Nil ^a^
*Echinops echinatus*	Nil ^a^	0 ^a^	Nil ^a^
*Eruca sativa* oil	58% ^d^	14 ^d^	52% ^d^
*Illicium verum*	38% ^a^	2 ^a^	35% ^a^
*Juglans regia* ^3^	51% ^b^	5 ^b^	50% ^b^
*Juglans regia* ^4^	52% ^b^	7 ^b^	51% ^b^
*Lagenaria siceraria* oil	Nil ^a^	0 ^a^	Nil ^a^
*Myristica fragrans* ^5^	39% ^a^	2 ^a^	35% ^a^
*Myristica fragrans* ^6^	35% ^a^	2 ^a^	32% ^a^
*Olea europaea*	28% ^a^	2 ^a^	31% ^a^
*Phyllanthus emblica* oil	Nil ^a^	0 ^a^	Nil ^a^
*Prunus dulcis* oil	Nil ^a^	0 ^a^	Nil ^a^
*Psoralea corylifolia* oil	Nil ^a^	0 ^a^	Nil ^a^
*Punica granatum*	55% ^a^	5 ^a^	52% ^a^
*Salvadora persica* ^7^	Nil ^a^	0 ^a^	Nil ^a^
*Salvadora persica* ^8^	Nil ^a^	0 ^a^	Nil ^a^
*Sesamum radiatum*	Nil ^a^	0 ^a^	Nil ^a^
*Syzygium aromaticum* oil	72% ^c^	16 ^c^	61% ^c^
*Terminalia arjuna*	41% ^a^	2 ^a^	38% ^a^
*Urtica dioica*	42% ^a^	2 ^a^	41% ^a^
*Wrightia tinctoria*	Nil ^a^	0 ^a^	Nil ^a^

^1^ Black garlic; ^2^ garlic; ^3^ stem peel; ^4^ root peel; ^5^ seed; ^6^ mace; ^7^ origin Saudi Arabia;^8^ Origin Pakistan; ^a^ = 200 µg/mL (max. tested concentration); ^b^ = 70 µg/mL; ^c^ = 0.19 µg/mL; ^d^ = 0.39 µg/mL; * *Pseudomonas. aeruginosa (*%); ** *Chromobacterium violaceum* (mm).

**Table 5:** Chemical composition of active plants according to literature.

Plant Species	Major Constituents
*Azadirachta indica*	Nimbidine, azadirachtin (Azadirachtin A), salannol, salannin [60]
*Eruca sativa*	Bis (4-isothiocyanatobutyl) disulphide (5000 µg/g), sulforaphane (743 µg/g), 2-phenylethyl isothiocyanate (158 µg/g), 3-butenyl isothiocyanate (259.6 µg/g), erucic acid (57%) [61]
*Illicium verum*	*Trans*-anethol (71.98%), feniculine (14.5%) [62] estragole (1.84%), limonene (1.38%), 4-allyl anisole (6.7%) [63]
*Juglans regia*	Juglone, regiolone (28.6%), proceroside (9.1%) [64] glansreginin B (11.5%), glansreginin A (10.4%) [65] quercetin, quercitrin, gallic acid [58]
*Myristica fragrans* ^1^	Myrislignan (22.59%), elemicin (13.99%), α-phellandrene (13.04%) [66] Sabinene (28%), β-pinene (9.72%), α-pinene (10.2%) [67] safrole, eugenol, caryophyllene, caryophyllene oxide, palmitic acid [68]
*Myristica fragrans* ^2^	Safrole, sabinene (28%), β-pinene (9.72%), eugenol, caryophyllene, caryophyllene oxide, palmitic acid [68]
*Syzygium aromaticum*	Eugenol (87–89%), caryophyllene (3.56%), eugenyl acetate (8.01%), α-humulene (0.04%), caryophyllene oxide (0.47%) [11,69]
*Punica granatum*	Gallic acid, caffeic acid, chlorogenic acid, ellagic acid, apigenin, quercetin, pelargonidin, cyanidin, punicalin, punicalagin, granatin A, granatin B [59,70]

^1^ mace; ^2^ seed.

**Table 6:** MIC (mg/mL) of pure compounds against clinical and reference strains.

Compound Name	*S. aureus* ^a^	*S. epidermidis* ^b^	*S. aureus* ^c^	*P. aeruginosa* ^a^
Juglone	0.0058	0.046	0.75	0.05
Caryophyllene-oxide	0.37	0.37	0.75	0.05
α-Humulene	0.023	0.37	0.75	0.187
Eugenol	0.0003	0.0003	0.039	0.019
2-Phenylethyl isothiocyanate	0.0058	0.023	0.093	0.18
Caryophyllene	>0.75	>0.75	0.75	0.14
Quercetin	>0.75	0.375	1.25	0.625
Gallic acid	>0.75	>0.75	0.75	0.75
Apigenin	0.25	0.125	0.125	0.25

^a^ Isolated strain; ^b^ isolated strain; ^c^ ATCC 33862; ^d^ ATCC 15442.

**Table 7:** Antibiofilm and anti-QS activity of tested compounds.

Name	Inhibition of Bacterial Biofilm ^a^	Inhibition of Bacterial Biofilm ^b^	Zone of Inhibition *	Violacein Inhibition
Juglone ^1^	0%	0%	1	20%
Caryophyllene-oxide ^2^	51%	17%	4	54%
α-Humulene ^3^	58%	56%	5	40%
Eugenol ^4^	55%	52%	15	57.2%
2-Phenylethyl-isothiocyanate ^5^	57%	56%	9	55.5%
Caryophyllene ^6^	0%	0%	5	50.8%
Quercetin ^7^	0%	52%	0	0%
Gallic acid ^8^	0%	0%	0	0%
Apigenin ^9^	48%	58%	0	0%

^1^ = 0.18 mg; ^2^ = 0.375 mg/mL; ^3^ = 0.62 mg/mL; ^4^ = 0.0.03%; ^5^ = 0.078%; ^6^ = 0.031 mg/mL; ^7−9^ = 1.2 mg/mL; * *Chromobacterium. violaceum* (mm); ^a^ *Staphylococcus aureus* isolated strain; ^b^ *Staphylococcus epidermidis* isolated strain.

## Supplementary Materials

The following are available online at https://www.mdpi.com/article/10.3390/antibiotics10121504/s1: Figure S1: HPLC-DAD analysis of *Punica granatum* (peel extract). Figure S2: HPLC-DAD analysis of *Juglon regia* (bark extract). Figure S3: HPLC-DAD analysis of *Juglon regia* (root extract). Figure S4: HPLC-DAD analysis of *Myristica fragrans* (mace extract). Figure S5: HPLC-DAD analysis of *Myristica fragrans* (seed extract). Figure S6: HPLC-DAD-MS-QToF analysis of *Juglans regia* (bark peel extract). Figure S7: HPLC-DAD-MS-QToF analysis of *Juglans regia* (root peel extract). Figure S8: HPLC-DAD-MS-QToF analysis of *Myrictica fragrans* (mace extract). Figure S9: HPLC-DAD-MS-QToF analysis of *Myrictica fragrans* (seed extract). Figure S10: HPLC-DAD-MS-QToF analysis of *Punica granatum* (peel extract). Figure S11: GC-MS chromatogram of *Syzygium aromaticum* oil. Figure S12: GC-MS chromatogram of *Eruca sativa* oil. Figure S13: GC-MS chromatogram of *Azadirachta indica* seed oil. Table S1: Primers sequencing parameters. Table S2: Optimized condition for polymerase chain reactions. Table S3: Optimized PCR conditions. Table S4: Identification of strains based on 16S rRNA gene sequence published in DNA database. Table S5: Identification of compounds from *Juglans regia* (bark peel *extract)* using HPLC-DAD-MS-QToF. Table S6: Identification of compounds from *Juglans regia* (root peel *extract)* using HPLC-DAD-MS-QToF. Table S7: Identification of compounds from *Myrictica fragrans* (mace *extract)* using HPLC-DAD-MS-QToF. Table S8: Identification of compounds from *Myrictica fragrans* (see *extract)* using HPLC-DAD-MS-QToF. Table S9: Identification of compounds from *Punica granatum* (peel extract) using HPLC-DAD-MS-QToF. Table S10: Docking score, H and non H-Bonding interactions of tested compounds.
