# Supplementary material for: Analysis of Plant Origin Antibiotics against Oral Bacterial Infections Using In Vitro and In Silico Techniques and Characterization of Active Constituents
Source: Antibiotics (Basel). 2021 Dec 8;10(12):1504. doi: 10.3390/antibiotics10121504 (PMC8699006; doi:10.3390/antibiotics10121504)
Supplement: Supplementary file 1 [file antibiotics-10-01504-s001.zip › antibiotics-1451806-supplementary.pdf]

Analysis of Plant Origin Antibiotics Against Oral Bacterial Infections Using *In vitro* and *In silico* Techniques and Characterization Of Active Constituents

Abdul Rafey [1], Adnan Amin [1], Muhammad Kamran [1], Uzma Haroon [2], Kainat Farooq [3], Kenn Foubert [4] and Luc Pieters [4,*]

[1] NPRL, Department of Pharmacognosy, Faculty of Pharmacy, Gomal University, D.I. Khan, Pakistan.; e-mail@e-mail.com; adnan.amin@gu.edu.pk (A.A.)
[2] Department of Dentistry, D.H.Q Teaching Hospital, D.I.Khan, KPK, Pakistan; e-mail@e-mail.com
[3] Sardar Begum Dental College, Ghandhara University, Peshawar, Pakistan
[4] Natural Products & Food Research and Analysis (NatuRA), Department of Pharmaceutical Sciences, University of Antwerp, Belgium; kenn.foubert@uantwerpen.be (K.F.); luc.pieters@uantwerpen.be (L.P.)
* Correspondence: adnan.amin@gu.edu.pk (A.A.); luc.pieters@uantwerpen.be (L.P.)

Supplementary Materials

DNA Extraction and 16S rDNA Sequencing

Briefly the bacterial colonies were collected Muller Hinton agar plates and placed in lysis solution (500 µL) in microtubule and incubated for 20-30 minutes at room temperature. This was followed by centrifugation for 3 minutes at 13000 rpm for phase separation. The supernatant was discarded while the pellet containing DNA was further processed (multiple washings with lysis solution). Pellet was again treated with 400 µl lysis solution, 13 µl of 20% SDS (Sodium Dodecyl sulphate) and 25 µl proteinase K. Samples were incubated at 37°C overnight.

The samples were treated further with 500 µl of phenol, chloroform and isoamyl alcohol (i.e. PCI solution). The suspended solutions were centrifuged at 13000 rpm for 10 minutes for gentle and through mixing. Aqueous phase was transferred to other tube for purification and separation of DNA. The aqueous layer was treated with 500 µl of chloroform and isoamyl alcohol (C:I, 24:1) and centrifuged again for 10 minutes at 13000

rpm. The aqueous layer was shifted into 1.5 ml centrifuge tube, 55 μl of sodium acetate and 500 μl of chilled isopropanol were added. Samples were incubated for 45 minutes at -20°C. Samples were centrifuged at 13000 rpm for 10 minutes. Supernatant was discarded and pellet was treated with 500 μl of 70% ethanol and centrifuged at 7500 rpm for 5 minutes in order to remove all impurities, pellet was kept while supernatant was discarded and air dried. DNA pellet was resuspended in TE Buffer (Tris EDTA) and stored at 4°C.

Table S1. Primers sequencing parameters

16SV3V4-F	CCTANGGGNNGCANCAG
16SV3V4-R	GGACTACNNGGGTATCTAAT TCCTCCGCTTATTGATATGC

Agarose Gel Electrophoresis:

Gel electrophoresis was performed using 1% agarose gel and the composition included 1 gram of agarose which was dissolved in 100 ml of 1X TAE buffer (Tris Acetic acid EDTA). Clear solution was formed after heating. 7 μl Ethidium Bromide was added in gel solution. Gel was poured into the gel casting tray with inserting combs. After solidification, gel caster was transferred to gel tank filled with 1X TAE buffer and combs were removed carefully. 2 μl of extracted DNA was mixed with 2 μl of 6X bromophenol blue dye (loading dye) and it was loaded in wells. The gel was run under specific parameters which included 500 mA of current with 75 volts for 35 minutes. Gel was visualized under UV Trans-Illuminator bio Doc Analyzer. Following gel picture is showing representative DNA bands with comparison to 1KB Ladder:

Polymerase Chain Reaction (PCR):

PCR is a molecular biology technique used to amplify a single copy or a specific sequence of DNA. 16SV3V4 primers were used to amplify the fungal samples. Sequences of forward and reverse primers are:

Following chemicals at provided concentrations were used:

- Template DNA
- Forward & reverse Primer (BGI Company)
- Taq polymerse enzyme 5U/ μL (Solis BioDyne FIREPol DNA polymerase, 01-01-00500)
- PCR buffer (Solis BioDyne FIREPol DNA polymerase, 01-01-00500)
- $MgCl_2$ (Solis BioDyne FIREPol DNA polymerase, 01-01-00500)
- dNTPs (Solis BioDyne, dNTPs Set, 02-21-00400)
- PCR water (Invitrogen RT PCR garde water, AM9935)

Table S2. Optimized condition for Polymerase chain reactions

**PCR Reagents**	**Stock Conc.**	**Working Conc.**	**Vol/Rec**	**Vol. x (n)**
DNA template	-	-	1 μL	
pF	10 μM	0.2 μM	0.4 μL	
Pr	10 μM	0.2 μM	0.4 μL	
DNTPs	10 mM	0.2 Mm	0.4 μL	
Buffer	10X	1X	2 μL	
$MgCl_2$	25 mM	2.5 Mm	2 μL	
taq Polymerase	5U/ μL	1.5 U	0.3 μL	
PCR H_2O			13.5 μL	
**Final Volume**			20 μL	

"n" would be any number for which you are making master mix.

Polymerase chain reactions were performed on a Galaxy XP Thermal Cycler (BIOER, PRC). Optimized PCR conditions were shown in table.

Table S3. Optimized PCR conditions

Steps	Sub-cycles	Conditions	PCR cycles
**Initial Denaturation**		95 °C, 10 min	1
**PCR Cycles**	Denaturation	95 °C, 1 min	40
	Primer annealing	54 °C, 1 min	
	Primer extension	72 °C, 1 min	
**Final extension**		72 °C, 10 min	1
**Hold**		04 °C, ∞	1

Table S4. Identification of strains based on 16S rRNA gene sequence published in DNA database.

S.No	Strain ID	Number of nucleotides of 16S rRNA gene	Closely related validly published taxa	Sequence accession number of closely related species	Similarity %age of 16S rRNA gene sequence with closely related species	No. of closely related species having >97% (>98%) similarity of 16S rRNA gene sequence
1.	U7(1)	402	Staphylococcus epidermidis (NCTC 11047(T)	UHDF01000003	99.75	>30
2.	U6	981	Staphylococcus aureus subsp. aureus (DSM 20231T)	AMYL01000007	99.39	6(5)

HPLC-DAD analysis

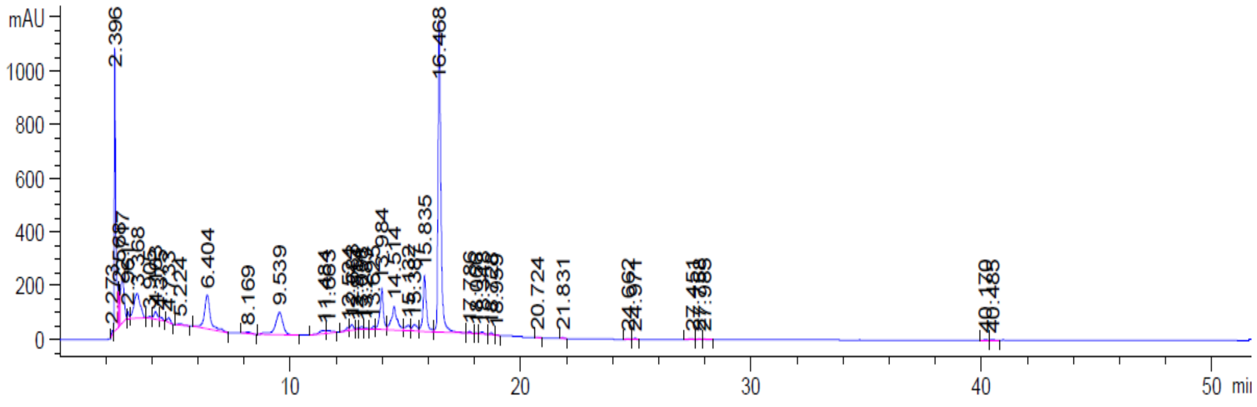

Figure S1. HPLC-DAD analysis of *Punica granatum* (peel extract)

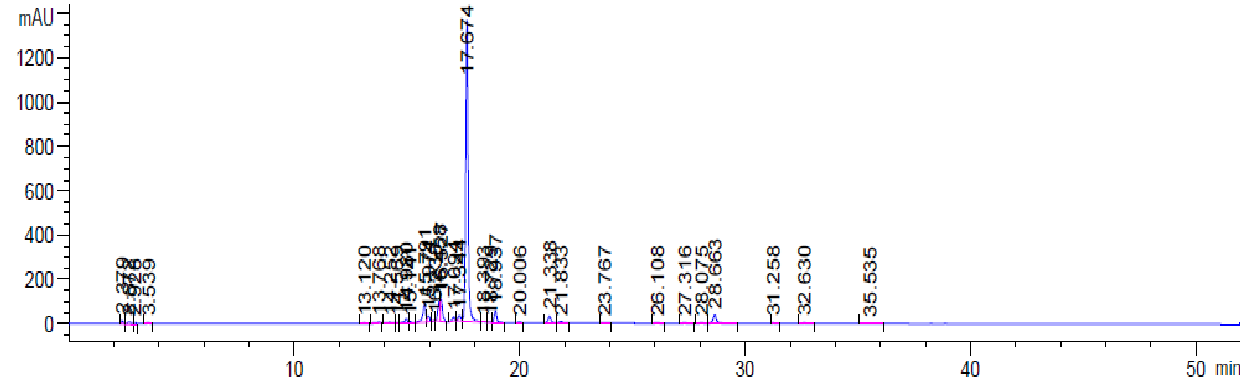

Figure S2. HPLC-DAD analysis of *Juglans regia* (bark extract)

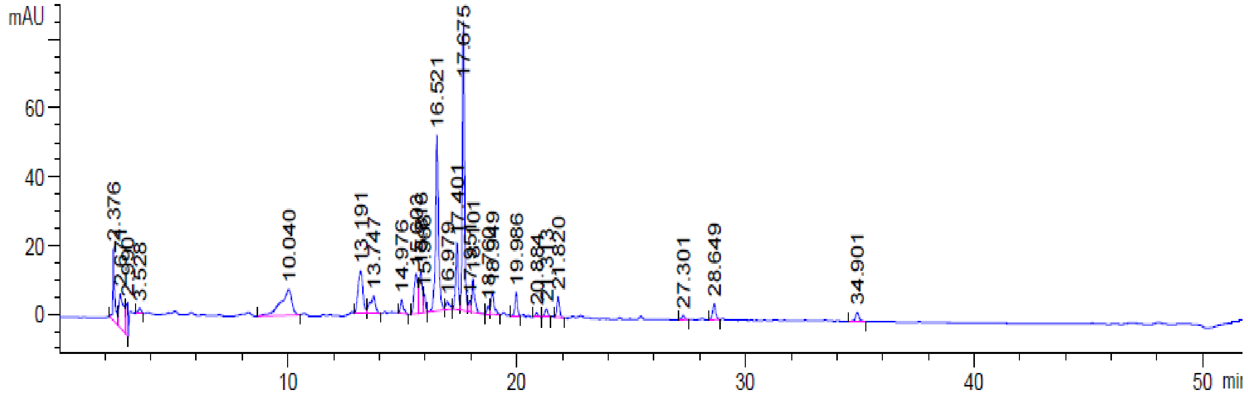

Figure S3. HPLC-DAD analysis of *Juglans regia* (root extract)

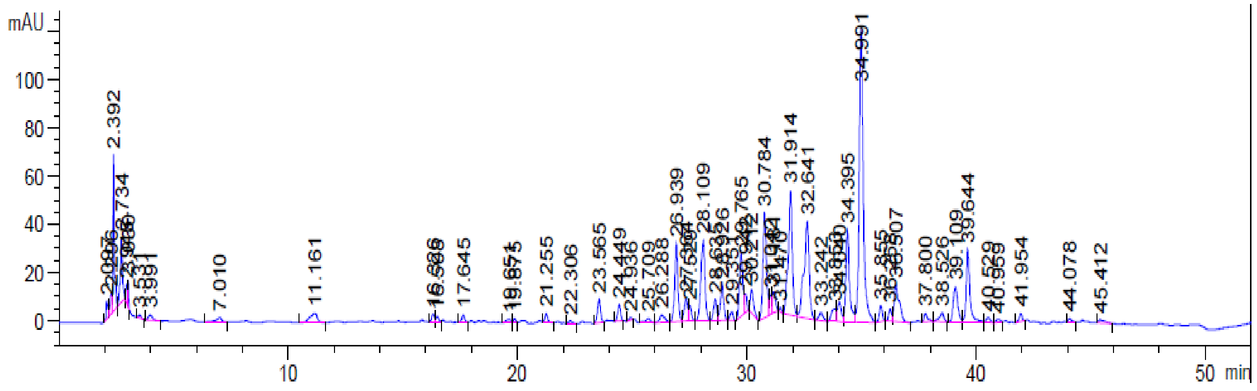

Figure S4. HPLC-DAD analysis of *Myristica fragrans* (mace extract)

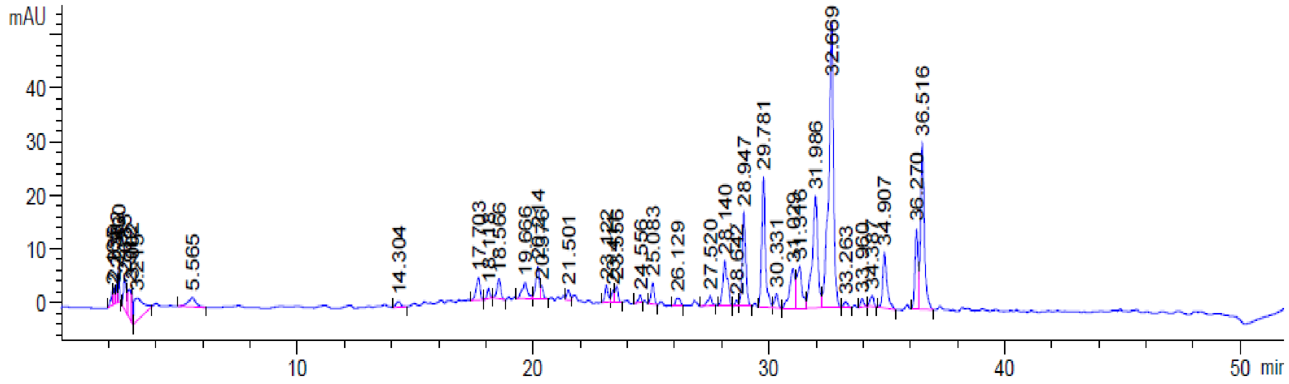

Figure S5. HPLC-DAD analysis of *Myristica fragrans* (seed extract)

HPLC-DAD-MS-QToF analysis

1. *Juglans regia* (bark peel)

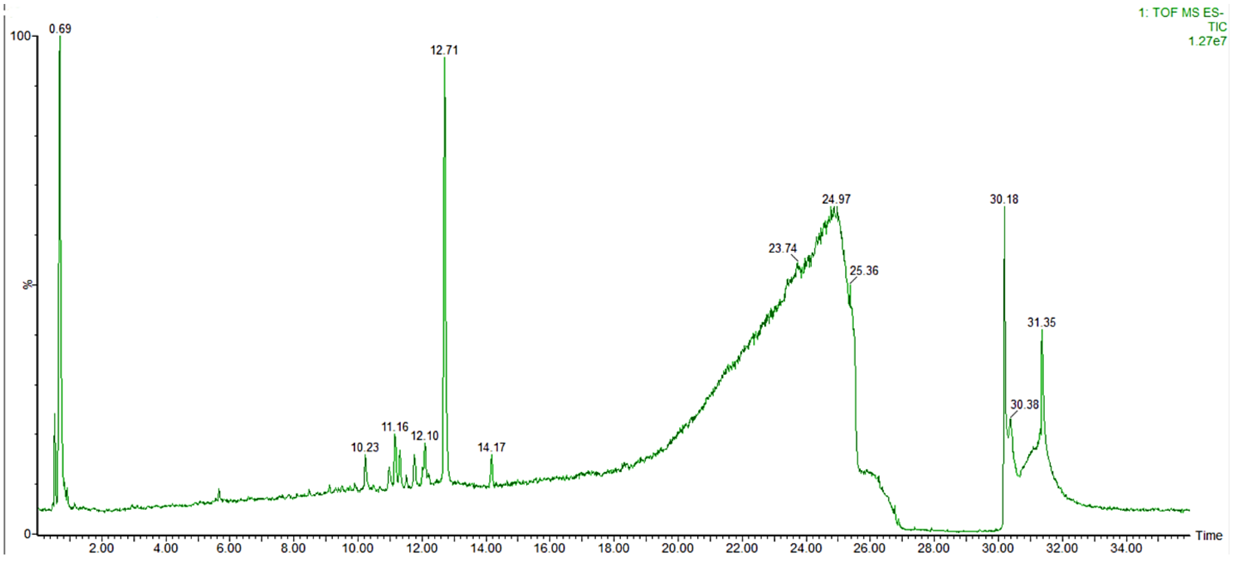

Figure S6. HPLC-DAD-QToF analysis is of *Juglans regia* (bark peel extract)

Table S5. Identification of compounds from *Juglans regia* (bark peel extract) using HPLC-DAD-QToF

Peak No	Compound identification	RT	$[M-H]^-$ (*m/z*)	MS/MS fragment signals (*m/z*)
1	Unknown	10.23	435.09	435.0912;<br>300.112; 303.0420;<br>285.0347<br>(Mass Bank)*
2	Quercetin-3-galactoside	11.16	463.08	463.0868; 461.0706;<br>300.0254; 301.0324;<br>271.0222; 255.124<br>(Mass Bank)*
3	Isoquercetin/ Quercetin-3-glucoside	11.30	463.08	463.0869; 461.0700;<br>300.0254; 301.0324;<br>271.0206;<br>(Mass Bank)*
4	Luteolin 7-glucoside	11.77	447.12	447.1263; 448.124;<br>285.075; 286.0712; 283.0145<br>(Mass Bank)*
5	Unknown	12.10	447.12	447.125; 448.1282;<br>433.0754;<br>300.0223; 302.0239;<br>285.075; 286.718<br>(Mass Bank)*
6	Quercitrin/ Quercetin 3-O-rhamnoside	12.71	447.09	447.0943; 445.0676<br>301.0405; 300.0405; 271.0196;<br>255.0280 (Rasu et al., 2020)
7	Unknown	14.12	431.09	431.069; 432.0990<br>285.0385; 284.0499<br>255.244 (Mass Bank)*

* Mass bank of North America; HMDB; SpectraBase Willey; massbank.eu

2. *Juglans regia* (root peel)

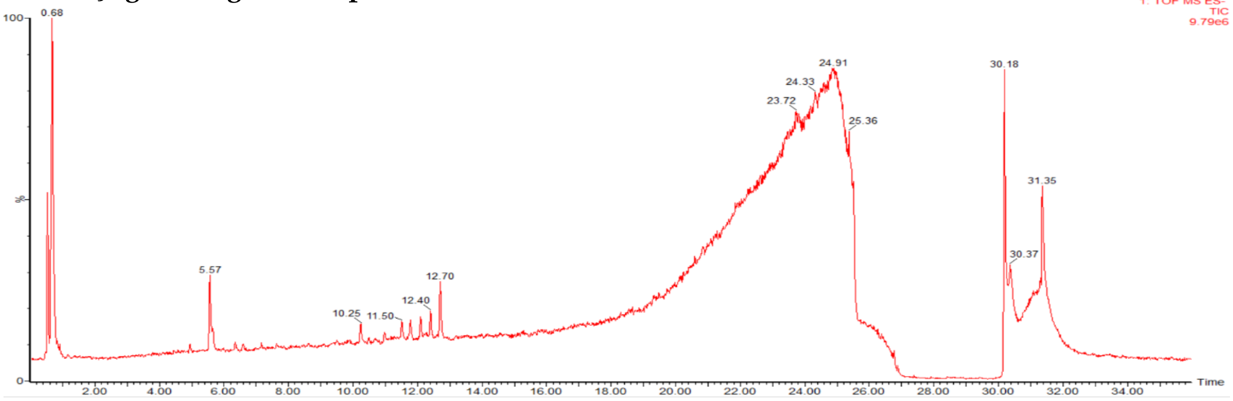

Figure S7. HPLC-DAD-QToF analysis is of *Juglans regia* (root peel extract)

Table S6. Identification of compounds from *Juglans regia* (root extract) using HPLC-DAD-QToF.

Peak No	Compound identification	RT	$[M-H]^-$ (m/z)	MS/MS fragment signals (m/z)
1	4-hydroxyphenyl-6-O-(4-hydroxy-2-methylenebutanoyl) glucopyranoside	5.57	369.1180	369.1180; 370.1220; 371.1208; 359.0921; 700.1441; 701.1432 (Rasu et al., 2020)
2	Unknown	10.25	435.092	435.0921; 436.0929; 361.1160; 285.0375
3	Unknown	10.98	435.0921	450.0903; 436.0818; 355.9374; 285.08331
4	Unknown	11.50	447.05	447.054; 448.0539; 315.0126; 299.9863
5	Luteolin 7-glucoside	11.77	447.12	447.1263; 448.124 285.075; 286.0712; 283.0145 (Mass bank) *
6	Unknown	12.10	447.12	447.125; 448.1282; 352.8524; 285.075; 286.718
7	Unknown	12.40	723.512	723.5016; 724.0502 725.51; 677.4947; 678.4974
8	Quercitrin/ Quercetin 3-O-rhamnoside	12.71	447.09	447.0943; 445.0676 301.0405; 300.0405; 271.0196; 255.0280 (Rasu et al., 2020)

* Mass bank of North America; HMDB; SpectraBase Willey; massbank.eu

3. *Myrictica fragrans* (mace)

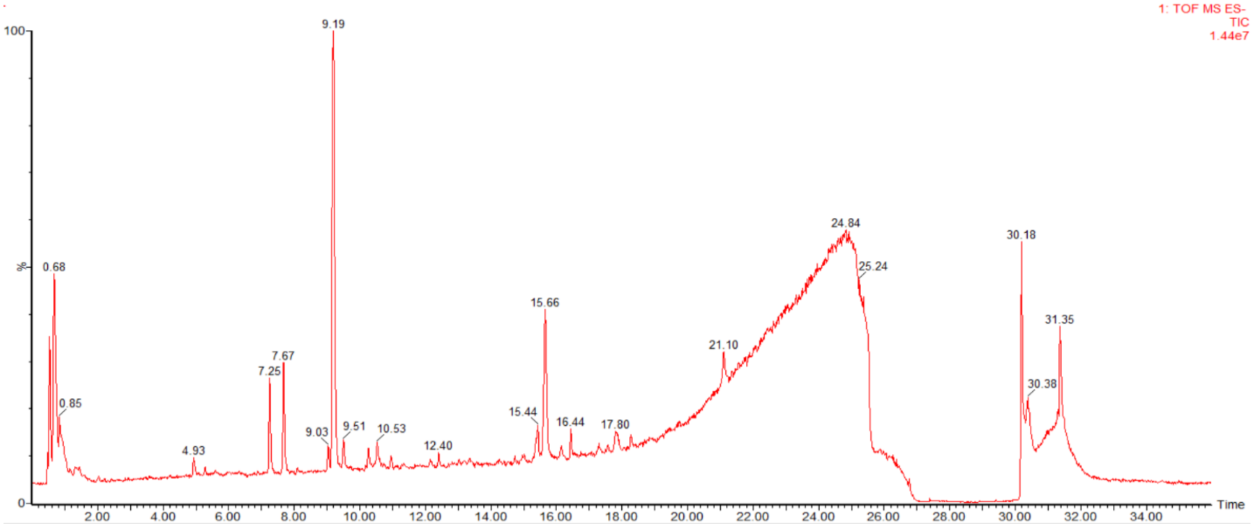

Figure S8. HPLC-DAD-QToF analysis is of *Myristica fragrans* (mace extract)

Table S7. Identification of compounds from *Myristica fragrans* (mace extract) using HPLC-DAD-QToF

Peak No	Compound identification	RT	[M-H]$^-$ (m/z)	MS/MS fragment signals (m/z)
1	Maceneolignan B	4.93	353	353.059; 354.075<br>233.0421;<br>205.0421<br>(Morikawa et al., 2016)
2	Unknown	7.25	519.17	519.1713;<br>473.16750; 509.1625;<br>311.113;<br>149.0588
3	Malabaricone-B	9.03	341.122	341.1225;<br>179.0637;<br>164.04<br>(Hou et al., 2012)
4	Myrisfrageal A	9.19	435.2	435.214; 311.1185;<br>149.0671<br>(Cao et al., 2013)
5	Unknown	9.51	455	311.1125;

				238.2541; 149.0594; 147.5478; 116.9277
6	Myrifralignan A	10.53	371.1	371.330; 145.92; 116.927; (Cao et al., 2015)
7	Unknown	12.40	713.2	713.2663; 505.207; 343.0152
8	Unknown	15.66	391.08	391.0828; 392.126; 343.1495; 241.0010

4. *Myristica fragrans* (seed)

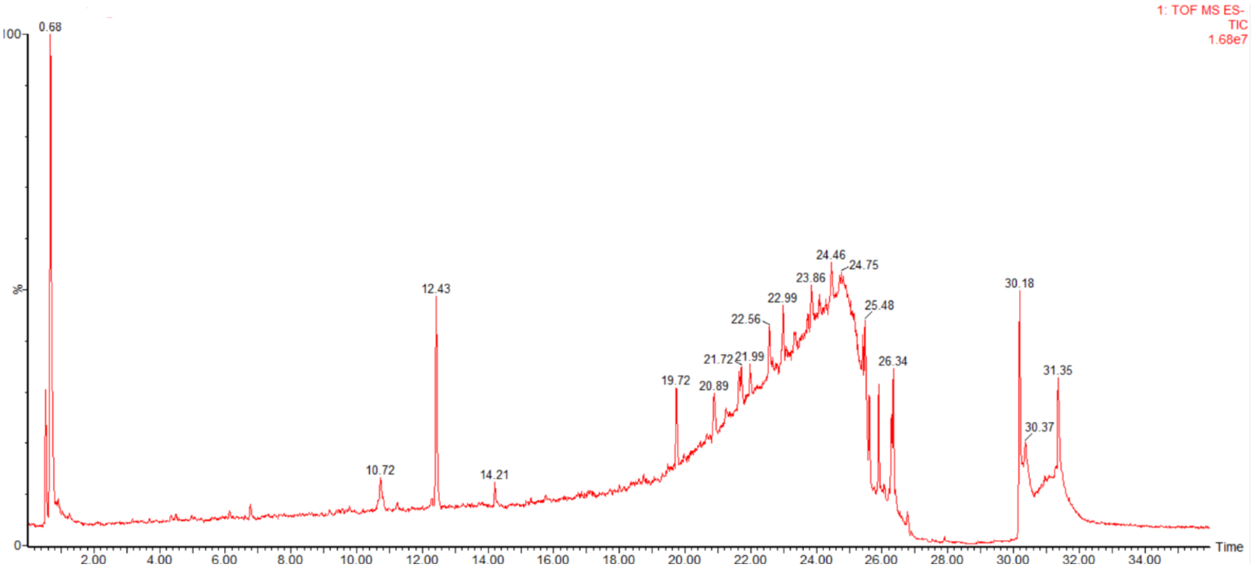

Figure S9. HPLC-DAD-MS-QToF analysis is of *Myristica fragrans* (seed extract)

Table S8. Identification of compounds from *Myristica fragrans* (see extract) using HPLC-DAD-QToF

Peak No	Compound identification	RT	$[M-H]^-$ (m/z)	MS/MS fragment signals (m/z)
1	Unknown	10.72	237.32	237.110; 116.231
2	Unknown	12.43	723.12	723.51; 677.5070
3	Unknown	14.21	949.6	949.1423; 939.637

5. ***Punica granatum* (peel)**

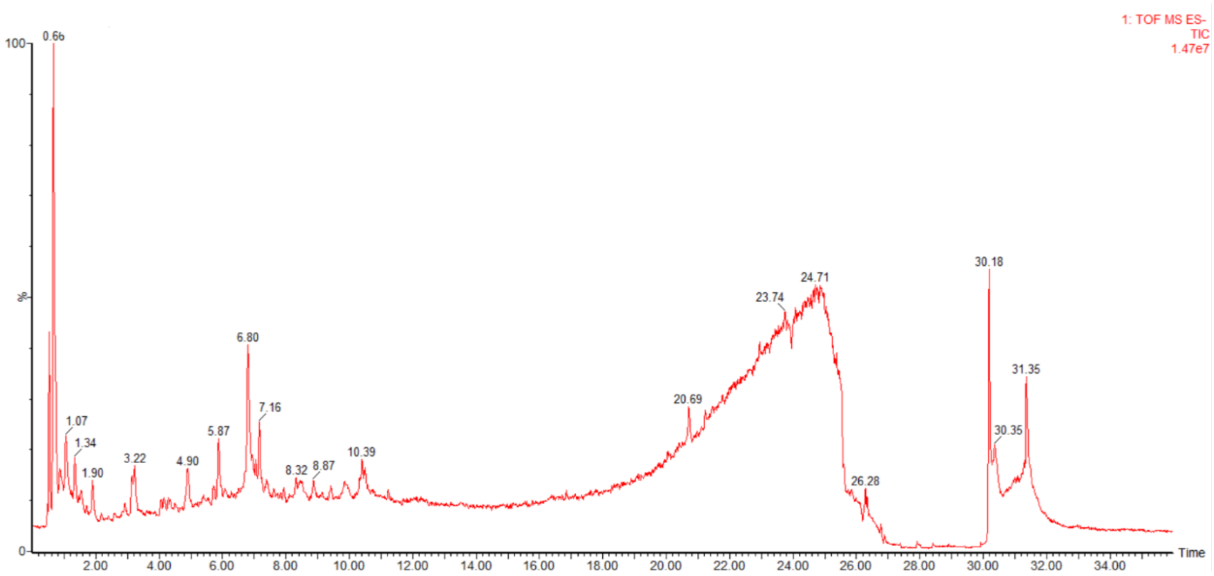

Figure S10. HPLC-DAD-QToF analysis is of *Punica granatum* (peel extract)

Table S9. Identification of compounds from *Punica granatum* (peel extract) using HPLC-DAD-QToF

Peak No	Compound identification	RT	$[M-H]^-$ (m/z)	MS/MS fragment signals (m/z)
1	hexahydroxydiphenoyl. – hexoside (HDDP-hexoside)	1.07	481.2	481.0613; 300.9963; 275.0167 (Mena et al., 2012; Hernández-Corroto et al., 2019)
2	hexahydroxydiphenoyl. – hexoside (HDDP-hexoside)	1.34	481.3	481.0611; 449.034; 300.9963; 275.0167; 247.0185 Hernández-Corroto et al., 2019)
3	Galloyl-hexoside	1.70	331.2	271.021; 211.0182; 169.998; 125.021 Hernández-Corroto et al., 2019)
4	Punicalin α	3.14	781.5	781.05; 721.029; 601.012 (Mena et al., 2012)
5	Punicalin β	3.22	781.23	781.05; 721.029; 600.9892; 449.009 (Mena et al., 2012)
6	Digalloyl-hexoside	4.14	483.04	483.046; 331.9964; 313.231; 169.004 (Mena et al., 2012)
7	di(HHDP-galloylglucose)-pentose	4.90	707.23	707.252; 541.183; 300.995; 301.235; 275.0125; 145.9632; 116.235 (Mena et al., 2012)

8	Pedunculagin I isomer	5.70	783.23	783.056;<br>721, 765;<br>481.0211;<br>300.125;<br>301.995<br>(Mena et al., 2012)
9	Punicalagin α	5.87	1083.2	781.012;<br>600.281;<br>301.231;<br>275.235<br>(Mena et al., 2012)
10	Punicalagin β	6.80	1083.2	781.06034;<br>600.99611;<br>301.231;<br>275.235<br>(Mena et al., 2012)
11	Galloyl-HHDP-hexoside	7.16	633.04	481.254;<br>463.123;<br>301.250;<br>275.124<br>(Mena et al., 2012)
12	Punicalagin isomer	8.87	783.1	481.0547;<br>300.9958;<br>298.9803;<br>274.9123<br>Hernández-Corroto et al., 2019)
13	Ellagic acid-deoxyhexoside	10.33	447.2	300.997;<br>270.9841<br>Hernández-Corroto et al., 2019

GC-MS Data

1. *Syzygium aromaticum*

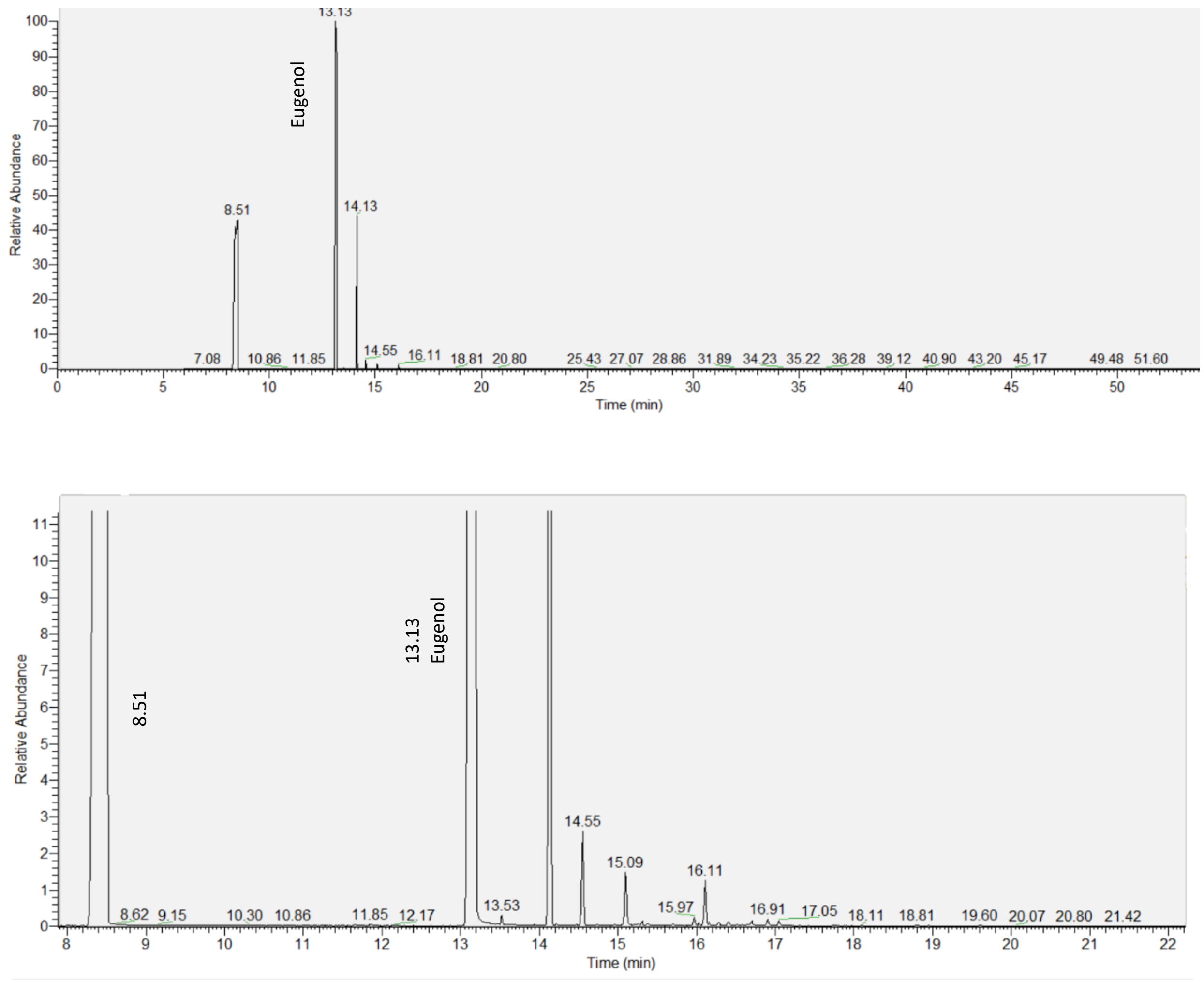

Figure S11: GC-MS chromatogram of *Syzygium aromaticum* oil

2. ***Eruca sativa***

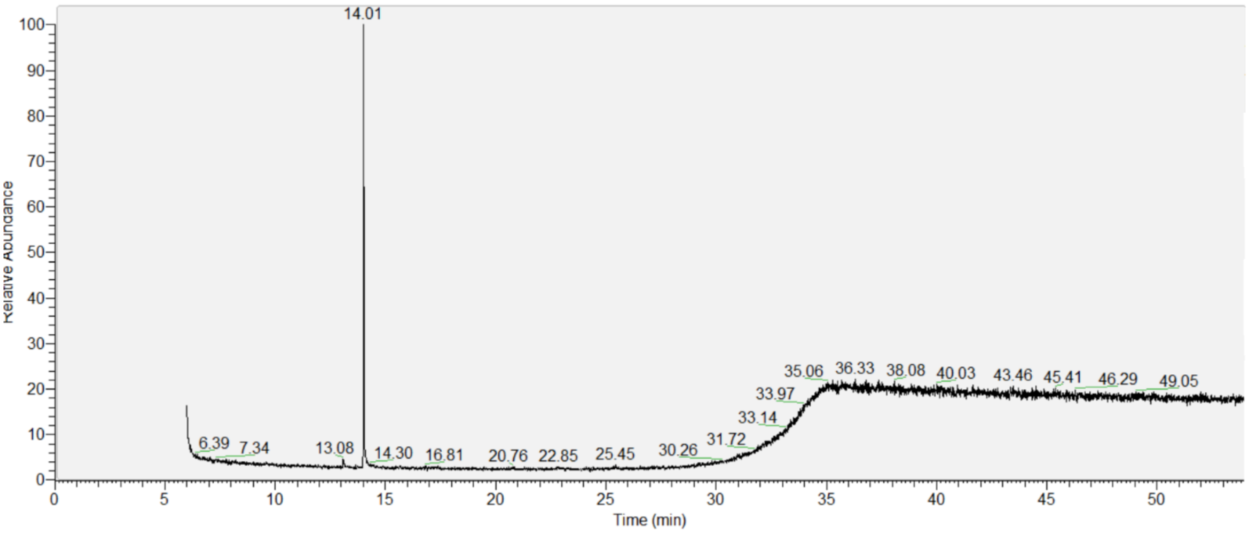

Figure S12: GC-MS chromatogram of *Eruca sativa* oil

3. ***Azadirachta indica***

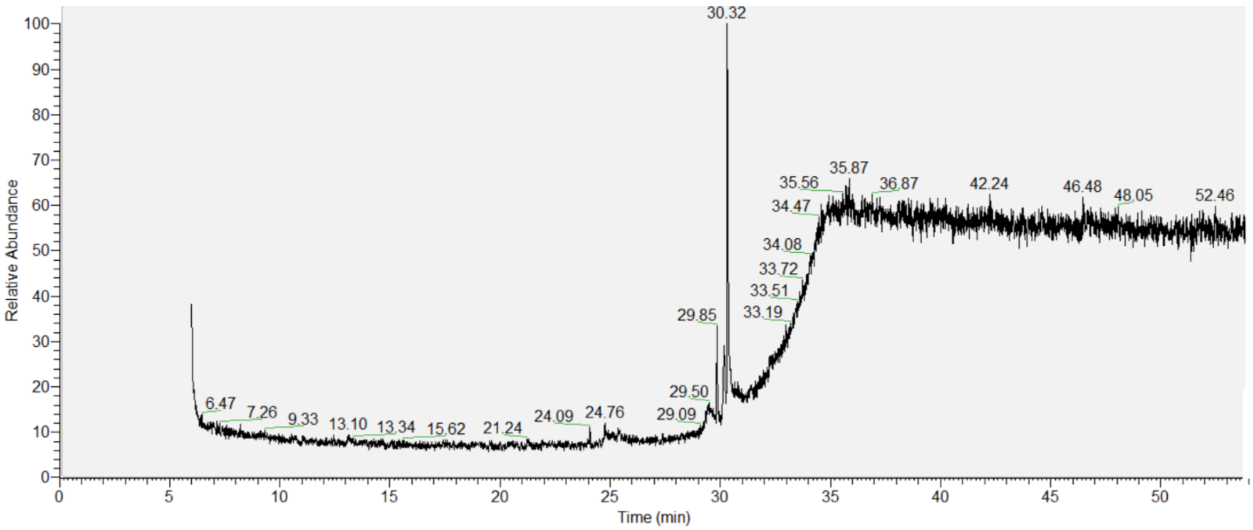

Figure S13: GC-MS chromatogram of *Azadirachta indica* seed oil

Table S10. Docking score, H and non H-Bonding interactions of tested compounds

Compound	Binding free energy ΔG (kJ mol^{-1})	Pose rank	No of H bonds	H Bond Interaction Residues	Other interaction residues
**2Q0J**					
Juglone	-8.1	1	5	Asp73, Asp 178, His221, His 282, Ser 285	Leu 277, Glu 182, Phe 195, Leu 193, Met286
Caryophyllene oxide	-5.7	1	1	Arg107	Ser102, Lys101, Trp100, Glu103, Asp130, Val106, Glu110, Trp129
- Humulene	-5.8	1	0	-	Leu277, Tyr 72, His71, Leu112, Leu 281, Arg 288.
Eugenol	-6.2	2	3	Asp73, His71, Asp178	Tyr72, His159, Leu193, Leu277 Ser273, His282, Ser285, Phe195
2-Phenylethyl isothiocyanate	-4.1	2	1	Ser294	Leu249, Gln252, Leu300, Pro299
Caryophyllene	-5.5	1	-	-	Leu202, Ala297, Leu298, Cys245 Leu242, Tyr238
Quercetin	-9.4	1	5	Glu182, Arg288, His71, Asp73, His221	His282, Leu277, Phe195, Asp178 Leu193
Gallic acid	-6.8	1	6	His282, Ser273, Glu182, His221, Asp73, Asp178	His159, Phe195, Leu193, Met286, Leu277, Phe276,
Apigenin	-9.0	1	3	Glu182,His221, Asp73	Leu193, His282, Leu277, Ser285 Arg288, His71, Asp178, Phe195
Quercitrin	-6.6	9	7	Asp259, Glu256, Gly255, Gln252, Ser294, Arg295 Ser257	Arg257, Leu355
**3QP1**					
Juglone	-5.6	3	4	Trp111, Glu112, Glu113, Gly138	Ser 137, Met110, Arg159, Gly158,

Caryophyllene oxide	-4.9	8	1	Arg114	Pro98, Ala118, Arg101, Glu123
- Humulene	-5.6	1	0	-	Leu12, Leu25, Leu24, Glu21, Pro13
Eugenol	-5.0	6	3	Trp111, Gly128, Gla112	Arg159, Gly158, Gy162, Arg163 Ser137, Met110
2-Phenylethyl isothiocyanate	-4.1	2	1	Arg163	Gly138, Met110, Ser137, Arg159, Glu112,
Caryophyllene	-5.8	1	-	-	Leu16, Leu25, Gln21, Leu12 Leu24, Pro13
Quercetin	-6.3	4	5	Asn116, Glu112,Trp111 Gly138, Gly158,	Glu113, Met110, Arg159, Ser137
Gallic acid	-5.1	1	7	Glu113, Glu112, Gly138, Trp111, Gly158, Arg163	Met110, Arg159, Ser137
Apigenin	-6.4	3	4	Glu112, Trp111, Gly128, Arg163	Arg159, Gly162, Ser137, Met110
Quercitrin	-7.2	4	6	Arg59, Gly136, Ser137, Pro52, Glu160, Arg163	Thr131, Gly134, Met135, Gly158 Ser53,

2Q0J (Structure of Pseudomonas Quinolone Signal Response Protein PqsE) 3QP1 (Crystal structure of CviR ligand-binding domain bound to the native ligand C6-HSL).
